# Management of Overweight during Childhood: A Focus Group Study on Health Professionals' Experiences in General Practice

**DOI:** 10.1155/2015/248985

**Published:** 2015-07-06

**Authors:** Lone Marie Larsen, Loni Ledderer, Dorte Ejg Jarbøl

**Affiliations:** ^1^Hans Christian Andersen Children's Hospital, Odense University Hospital, 5000 Odense C, Denmark; ^2^Department of Public Health, Aarhus University, 8000 Århus C, Denmark; ^3^Research Unit of General Practice, Department of Public Health, University of Southern Denmark, 5000 Odense C, Denmark

## Abstract

*Background*. Because of the increasing prevalence of overweight and obesity in childhood in the Western world, focus on the management in general practice has also increased. *Objective*. To explore the experiences of general practitioners (GPs) and practice nurses participating in a randomised controlled trial (RCT) comparing two management programmes in general practice for children who are overweight or obese. *Methods*. Three focus groups with GPs and nurses participating in the RCT. Transcribed data were analysed using systematic text condensation followed by thematic analysis. *Results*. Health professionals considered it their responsibility to offer a management programme to overweight children. They recognised that management of overweight during childhood was a complex task that required an evidence-based strategy with the possibility of supervision. Health professionals experienced a barrier to addressing overweight in children. However, increasing awareness of obesity in childhood and its consequences in society was considered helpful to reach an understanding of the articulations concerning how best to address the issue. *Conclusions*. Health professionals in general practice recognised that they have a special obligation, capacity, and role in the management of obesity in childhood. Implementation of future management programmes must address existing barriers beyond an evidence-based standardised strategy.

## 1. Introduction

Overweight and obesity in children are conditions that are increasingly common in the Western world, including Denmark [1, 2], with significant adverse effects on physical and psychosocial health in childhood [3, 4]. Proactive strategies during childhood have been advocated to support prevention of overweight and obesity [5, 6]. As a large majority of overweight preschool children have the first point of contact in primary health care, international attention has focused on the treatment of obesity in childhood by primary care physicians. In Danish primary health care, the Preventive Child Health Examinations (PCHE) during the first 5 years of life provide an opportunity to detect overweight in children [7] and to offer an early and economically feasible management programme when needed.

In a randomised trial performed from August 2007 to November 2010 in 60 general practices in Denmark, a total of 80 overweight children aged 5–9 years and their families undertook either a programme with health consultations in general practice during a 2-year period or a more complex programme including lifestyle education performed by dieticians, physiotherapists, and psychologists for the children and their families, in addition to health consultations in general practice. The study investigator visited all participating GPs, informed them about the study, and introduced them to a lifestyle intervention strategy using a “small steps and realistic goals” approach. They also received written material on the study strategy and the lifestyle intervention strategy, and a chart was used at monthly consultations in general practice. The two strategies' effects on weight loss did not differ significantly. About 2/3 of the children in both groups lost weight, and the majority of participants in both groups in the randomised controlled trial (RCT) continued to participate in the study beyond 1 year. The RCT is described in detail elsewhere [8].

Health professionals (HPs) are presumably central to the successful implementation of programmes to treat overweight and obesity in childhood; however, little is known about their views on the implementation of the new strategy in general practice. In order to implement effective management programmes for overweight children, knowledge is needed regarding the experience of such programmes. Investigations of barriers to and facilitators of recruitment of overweight children and management of children who are overweight or obese in general practice can contribute to the existing knowledge about the management of overweight and obesity in childhood.

The aim of this qualitative focus group study was to explore the experiences of GPs and practice nurses as they participated in an RCT that compared two management programmes in general practice for overweight and obesity in childhood, with a specific focus on the barriers to and facilitators of programme implementation.

## 2. Materials and Methods

For this study we used a qualitative design. Between June 2010 and April 2011, we conducted focus group interviews in order to gain understanding of HPs' experiences with a management programme in general practice for overweight and obesity in childhood [9, 10]. Each interview was facilitated by the same moderator (Dorte Ejg Jarbøl) and observed by the same researcher (Lone Marie Larsen). All participants received written information, including a statement that participation was voluntary and the results of the study would be anonymous.

### 2.1. Participants

Eight GPs and six practice nurses (subsequently referred to as HPs) participated in three focus group interviews. The participants were invited by selecting the general practices that had obtained experience in the management of children who are overweight or obese through inclusion of at least one child in the RCT, further ensuring that the sample consisted of participants with different gender, profession, and clinical experience. The characteristics of the focus group participants are presented in Table 1.

### 2.2. Focus Group Interviews

We presented ourselves as colleagues and researchers, and the participants were encouraged to speak freely and to raise issues of importance to them. We asked participants to reflect on the introductory questions presented as follows.

The topics for discussion during the focus group interview were as follows:

 Barriers and facilitators to identifying obesity in childhood at the Preventive Child Health Examinations and motivation for inclusion in the RCT:
  - achieving a long-term treatment process with the family in the RCT,
  - the dialogue about diet and physical activity,
  - the dialogue about prevention of lifestyle-related diseases.
 Considerations on the management of overweight children and families in general practice.
 Considerations about municipal and hospital collaborative relationships with general practice concerning overweight children and their families.

Various ideas and issues were pursued as they emerged during the interviews, and the participants were also allowed to go beyond the themes. Each focus group interview lasted 60–70 minutes.

### 2.3. Data Analysis

The interviews were audio taped and transcribed verbatim. The transcripts were used for analysis, and all authors analysed the text independently. The text units were coded; we categorised statements based on the introductory questions, using a systematic text condensation inspired by Strauss and Corbin [11]. The basic idea was to find barriers to and facilitators of the management programmes brought up in discussion by the participants [12]. The content in each group was compared with the other groups to grasp diverse aspects of the participants' experiences. We identified the text units that summarised the discussions on including and retaining children and their families in a childhood weight management strategy. After comparing the results, differences were discussed to ensure consensus and that all data were appropriately categorised. We then identified three main categories that were catalytic for the management of obesity in childhood and the responsibility of HPs in general practice.

## 3. Results

The results included HPs' views on management and communication with children and families in a new programme addressing obesity in childhood. There were three categories: (1) management of children who are overweight or obese in general practice, an important but difficult task; (2) communication and relationship with parents about overweight and obesity; and (3) sustaining motivation in a long-term programme for obesity in childhood. The categories are elaborated, with specific focus on the facilitators of and barriers to the programme.

### 3.1. Management of Children Who Are Overweight or Obese in General Practice: An Important but Difficult Task

The HPs perceived that general practice had an important task and a certain responsibility to offer a management programme to overweight children and their families. The HPs drew a parallel to the treatment of other diseases in general practice; they perceived that detection of overweight was the purpose of weight measurement at the PCHE and that it was obvious that a programme should be offered if the child was diagnosed as overweight.

*It goes without saying, the way I see it – well, just as you would measure blood pressure in a 60-year-old, who comes to the clinic and says “Can I have my blood pressure measured?” – well it's just as natural that we catch them at the 5-year health examination, it's obvious. (GP)*

The HPs found that even though they thought that they had a special obligation, managing overweight and obesity in childhood was a demanding, complex task that required lots of skill, time, patience, and persistence from both the HPs and the family. Some HPs were concerned about their own ability to address overweight in their patients, especially when they expected that the family's response would be negative. The HPs might be afraid to lose the family's trust and that the family might then want to change to another general practice, with a loss of continuity of care as a result.

*Well I don't find it easy to broach the subject of their children being overweight. I certainly don't think it's easy. When the parents don't mention it themselves. (GP)*

### 3.2. Relationship with Families

The families expected the GP to address children who were overweight or obese, because of their role as the family physician. Usually a GP has valuable knowledge of the family history and dynamics, because a GP is regularly in contact with individual family members and often has a close relationship with the entire family. The HPs believed that families benefitted from a special patient-clinician relationship and that it provided stability and continuity in contact.

*Yes, but who else should do it, if not the family doctor? (GP and nurse)*

The possibility of ongoing and stable contact between general practice and the entire family was seen as an advantage in performing a long-term management programme that targeted obesity in childhood. Furthermore, the GP was able to profit from an established relationship that had been ongoing for several years. Thus, it was also possible to closely follow and register whether families that discontinued programme participation earlier might be motivated to rejoin a programme later. Children who had participated in a weight management programme previously might also be more inclined to ask for help again, when they grew older and probably had obtained more insight into the condition. During the interviews, HPs communicated the concern that discussing what was often seen as a sensitive topic might adversely affect the clinician-patient relationship.

*But of course it's very much a question of tact and a balancing act and well …well at least you have to tread carefully. It also depends on how good a contact you already think you have. (GP and nurse)*

### 3.3. Appreciation of Guidelines

The HPs thought that it was important to have tools and guidelines to identify the families who would benefit from the programme and to guide the treatment of the child and offer support to the family. The HPs tended to value the guidelines and concrete strategies used in the programmes.

*But it is also extremely positive that there are any offers at all, because you may feel you are totally at a loss, if you are only dealing with overweight children. It is terrific that we have had the possibility of doing something and have had some sort of support (the project). (GP)*

As part of the programme, HPs had the opportunity to have supervision. They were able to receive feedback, ask questions, and discuss difficult aspects of treatment. The option of supervision was appreciated and considered important.

*Some kind of supervision, I think so too. That was also your experience, wasn't it? A lot of benefit from supervision. (Nurse)*

HPs appreciated the opportunity to receive feedback from peers and to share experiences with each other. They were also interested in guidelines and evidence-based knowledge about the subject.

*We need knowledge about when our efforts have the largest effects. When do we start? We need clear guidelines on how to get the greatest effect/what really works. (GP)*

Concrete instructions were preferred in daily consultations, and HPs specifically asked for knowledge about “what works.” However, they did recognize that management of children who are overweight or obese was complicated and difficult to fit into a strict guideline.

### 3.4. Lack of Time

There was concern about the extra time and energy required to manage such a task in general practice. HPs thought that this might be a barrier to broaching the topic during busy periods.

*Because I think that it is so difficult, and I think that we undertake a bigger role that we are really capable of tackling. Well, having the resources for it, right? (GP)*

### 3.5. Communication with Parents about Children Who Are Overweight or Obese

Communication is an important part of treating overweight. HPs identified several barriers and facilitators in communication with parents about overweight. Issues concerned their willingness to discuss the topic, the parents' response to their child being assessed as overweight, and the HPs' discomfort in addressing the condition. The increasing public awareness of overweight and its consequences was experienced as a facilitator to addressing the topic appropriately at the PCHE. Several parents were aware of the issue, and some parents did expect to receive some kind of help if the child was diagnosed with overweight. The HPs experienced that some parents did not recognise their child as being overweight, responded with denial, and questioned the need of an intervention.

*But where they say, well she (child) is just a bit stocky, you know the usual – he or she is just heavily built. They do not seem to recognise that the child is overweight. (GP)*

HPs perceived that some parents felt that they were being accused when they were confronted with information about children who were overweight, while other parents reacted with guilt about letting the child become overweight. Parents' reactions were often guided by emotion, and HPs experienced such emotional situations as a challenge and a threat to the continuing success of the clinician-patient relationship. When confronted with the topic of overweight, some parents did not want the HPs to interfere. They expressed doubts about the effectiveness of a programme and wanted to address the condition themselves. Other parents appreciated that the HPs cared about their children and talked about weight issues and were also able to offer a programme that might help. Parents with personal experience of consequences of overweight during childhood were experienced as facilitators, both with regard to the introduction of the topic and in supporting adherence to a long-term management programme.

HPs expressed feeling a need for a “strong” family with a stable life situation in order for a lifestyle intervention programme to be successful. If the HPs observed significant social or psychological challenges in the family, they tended to say nothing about overweight.

### 3.6. Sustaining Motivation in a Long-Term Management Programme

It is important to sustain motivation and interest in the program in order for it to succeed, and there are several aspects to motivation. An important element was the family agreeing on the need to participate in a programme that targeted obesity in childhood. It was experienced as a facilitator when the family itself demanded an intervention at the PCHE. Families persuaded to participate in a programme were less likely to succeed in lifestyle changes and were more likely to discontinue the programme early.

*The thing is that we know them on a continuous basis and see them in many different contexts. Once we have gained their trust, that is what we rely on in an awful lot of contexts, right? The thing is that they trust us, and that is absolutely vital…in our capacity. Well, that is … that is totally crucial. (GP)*

Trustworthiness and an ongoing relationship between the family and general practice were considered important elements in maintaining the families in a long-term programme to manage children who were overweight or obese.

According to the HPs' experiences, the main elements in sustaining motivation during a long-term management programme were stabilising the child's weight through the programme, positive experiences during the process (e.g., in lifestyle changes bringing new joy to the family), and acknowledgement from the surroundings for managing to implement new lifestyle strategies.

*It gives great pride/satisfaction to all parties, when it succeeds (i.e., for the child, parents and staff in general practice). And that is the motivating factor, when you…. And then stick to the good development and ask, what really made this happen? What succeeded? (GP)*

There was a common understanding among HPs that stable psychological, social, and economic conditions and stable life situations in the family were crucial to achieving successful long-term participation in the programme.

*They are also faced with a lot of other stuff, which we don't… well, like divorce and problems with dad and problems with mum. So sometimes one does understand that this sort of comfort eating, if you are sort of disposed to it. The really big problem getting this to succeed is that one has stable family relations. (GP)*

In the RCT, one management strategy included “lifestyle education” performed by experts, in addition to health consultations in practice. According to the HPs' experiences some families experienced this education outside general practice as a facilitator, while others experienced it as a barrier. Not all families found it beneficial to obtain input from experts and meet other families in the same situation. HPs suggested an opportunity for referral to an optional external lifestyle educational programme. Finally, HPs thought that their own positive experiences from the RCT would act as a facilitator in their future management of obesity in childhood in general practice.

## 4. Discussion

HPs in general practice recognised that they had a special obligation, capacity, and role in the management of children who are overweight or obese. However, they experienced a number of barriers: their own perception of family resources, their understanding of the situation, communication with parents about the child being overweight, and sustaining motivation during a long-term management programme.

These results are in line with findings from several other studies [13–15]. In one study GPs felt optimistic and believed they were able to make a difference [14]. In another, the GPs undertook a key role in promoting a trustful setting in general practice. It provided them with the opportunity to intervene early in obesity in childhood and a positive relationship with the families [16]. Managing children who are overweight or obese is far more complex than management of adult obesity, which is also considered a complex issue [17], because the HP must promote healthy weight not only to the child, but also to the whole family [18]. As Edmunds [19] also found, the most successful consultations and programme management occurred when the HP and parents had a positive attitude towards each other and a common understanding of the issues involved. The overall experience in our study was that parents were generally interested in participating in management of overweight, once the subject was raised.

Having a concrete strategy to follow, as provided by the programme in the RCT and the possibility of using an external expert, appeared to help the HPs to meet the challenges. The successes were agreement on the need for an intervention, positive experiences during the process, and stabilisation of the patient's weight. These issues were also regarded as important factors in other studies [15, 16]. However, an English study concluded that GPs believed that obesity was not within their professional domain, even though patients in that study wanted their GP to address it [20]. Pocock et al. [18] point to the fact that increasing awareness and concern about childhood obesity and its consequences in society must go beyond general practice and involve the local environment.

### 4.1. Implications for Clinical Practice

The assessment of obesity in childhood and appropriate management programmes pose a challenge to general practice. Time constraints, lack of training, and lack of resources in general practice concerning the specific issue must be taken seriously during the implementation of future interventions. Although general practice has an opportunity to offer management of children who are overweight or obese, overweight, evidence-based management strategies are required. Although HPs in general practice were prepared to implement a programme to manage overweight, supervision and training were essential to be able to manage this new professional task. The physician-patient relationship is highly appreciated and appeared to influence the implementation process. These personal and emotional aspects of overweight go beyond evidence-based guidelines and must be taken into account in future training of HPs.

### 4.2. Strengths and Limitations

The present study used a qualitative design with open-ended questions to encourage discussion in the group sessions. We used this method to explore the collective perception negotiated within groups, and therefore we did not obtain an in-depth understanding of each individual HP's attitude and perception of managing overweight or obese children in general practice. We asked the 14 participants to bring forth their own experiences from participating in an RCT in general practice that targeted obesity in childhood. Participants were recruited among HPs who succeeded in implementing the programme for overweight children and their families. Individual characteristics of the GPs and nurses, such as age, gender, BMI, and their own lifestyles, might have influenced their interest in the program, the implementation process, and how the new program is offered to the families. One study suggests that physicians with a normal BMI are more likely to provide obesity care [21]. The fact that all focus group participants except one were women mirrors the gender split of participants in the RCT and underscores the fact that an overwhelming proportion of nurses in Denmark are women. The small sample size may have resulted in a positive attitude towards the overweight programme. In a study of obese adults, the authors point to the importance of having an active interest in overweight [22]. However, the results provided an understanding of the challenges that HPs face in everyday work with overweight. We assume that the attitude of HPs and commonality of obesity in childhood might be comparable to general practices in Western societies. The results also add to the existing literature on overweight. Our finding may contribute to a more nuanced understanding of the pros and cons of weight management programmes in general practice.

## 5. Conclusion

Our findings indicate that HPs in general practice are concerned about their professional role and obligations in the management of obesity in childhood. Although they had difficulties working with childhood obesity, they appreciated taking part in the study and exploring new ways of handling the topic. The intervention was acknowledged as promoting the implementation of new tasks. However, the implementation of future management programmes must address existing barriers beyond an evidence-based standardised strategy.

## Figures and Tables

**Table 1:** Characteristics of the focus group participants.

Participants	Number	Gender (*N*)		Work in GP	Location	
		Female	Male	Years (*N*)	Urban	Rural
General practitioner	8	7	1	3–15	3	5
Nurse	6	6	0	3.5–15	3	3
